# Phase I trial of volasertib, a Polo-like kinase inhibitor, plus platinum agents in solid tumors: safety, pharmacokinetics and activity

**DOI:** 10.1007/s10637-015-0223-9

**Published:** 2015-03-22

**Authors:** Ahmad Awada, Herlinde Dumez, Philippe G. Aftimos, Jo Costermans, Sylvie Bartholomeus, Kathleen Forceville, Thierry Berghmans, Marie-Anne Meeus, Jessica Cescutti, Gerd Munzert, Korinna Pilz, Dan Liu, Patrick Schöffski

**Affiliations:** 1 Medical Oncology Clinic, Institut Jules Bordet, Université Libre de Bruxelles, Boulevard de Waterloo 121, B-1000 Brussels, Belgium; 2 Department of General Medical Oncology and Laboratory for Experimental Oncology, Department of Oncology, University Hospitals Leuven and KU Leuven, Gasthuisberg, Herestraat 49, 3000 Leuven, Belgium; 3 SCS Boehringer Ingelheim Comm. V, Avenue Ariane 16, 1200 Brussels, Belgium; 4 Boehringer Ingelheim, 12 Rue André Huet, 51100 Reims, France; 5 Boehringer Ingelheim Pharma GmbH & Co. KG, Birkendorfer Street 65, 88400 Biberach, Germany

**Keywords:** Polo-like kinase inhibitor, Solid tumors, Phase I trial, Volasertib, Platinum therapy

## Abstract

*Background* This trial evaluated the maximum tolerated dose (MTD), safety, pharmacokinetics, and activity of volasertib, a selective Polo-like kinase 1 inhibitor that induces mitotic arrest and apoptosis, combined with cisplatin or carboplatin in patients with advanced/metastatic solid tumors (NCT00969761; 1230.6). *Methods* Sequential patient cohorts (3 + 3 dose-escalation design) received a single infusion of volasertib (100–350 mg) with cisplatin (60–100 mg/m^2^) or carboplatin (area under the concentration versus time curve [AUC]4–AUC6) on day 1 every 3 weeks for up to six cycles. Sixty-one patients received volasertib/cisplatin (*n* = 30) or volasertib/carboplatin (*n* = 31) for a median of 3.5 (range, 1–6) and 2.0 (range, 1–6) treatment cycles, respectively. *Results* The most common cycle 1 dose-limiting toxicities (DLTs) were thrombocytopenia, neutropenia and fatigue. MTDs (based on cycle 1 DLTs) were determined to be volasertib 300 mg plus cisplatin 100 mg/m^2^ and volasertib 300 mg plus carboplatin AUC6. Co-administration did not affect the pharmacokinetics of each drug. Partial responses were observed in two patients in each arm. Stable disease was achieved in 11 and six patients treated with volasertib/cisplatin and volasertib/carboplatin, respectively. *Conclusions* Volasertib plus cisplatin or carboplatin at full single-agent doses was generally manageable and demonstrated activity in heavily pretreated patients with advanced solid tumors.

**Electronic supplementary material:**

The online version of this article (doi:10.1007/s10637-015-0223-9) contains supplementary material, which is available to authorized users.

## Introduction

Most advanced or metastatic solid tumors are incurable despite the availability of multiple treatment modalities such as surgery, cytotoxic drugs, radiation therapy, and combinations of these therapies. Response to treatment in the advanced setting is dependent on the tumor type and treatment modality; however, these responses are rarely long lasting and are often followed by tumor progression and subsequently death. Novel treatment approaches are therefore required.

Polo-like kinase (Plk) 1 is a key enzyme regulating essential steps of mitosis including mitotic entry, centrosome maturation and separation, formation of the bipolar spindle, transition from metaphase to anaphase, and initiation of cytokinesis [1,2]. The functional relevance of Plk1 has been demonstrated in vitro by ‘knock-down’ experiments in cancer cell lines. In these experiments, depletion of Plk1 was accompanied by cell cycle arrest and apoptosis [3–6]. Overexpression of Plk1 has been observed in multiple human cancers [6–10] and has been associated with poor prognosis [11]. These findings suggest that Plk1 may be a promising target in cancer.

Volasertib, an investigational agent, is a potent and selective cell cycle kinase inhibitor that induces mitotic arrest and apoptosis by targeting Plk at low nanomolar concentrations [12]. Volasertib selectively inhibits Plk1 and, to a lesser extent, two other members of the Plk family, Plk2 and Plk3, but does not inhibit unrelated kinases (>50 other kinases) at concentrations up to 10 μM [12]. Compared with BI 2536 (the first Plk inhibitor to be developed by Boehringer Ingelheim), volasertib showed a high volume of distribution, indicating good tissue penetration, and a long terminal half-life (t_1/2_) in preclinical studies [12]. As a result, clinical investigation of BI 2536 was halted and clinical development continued with volasertib. Early clinical data has indicated that the adverse event (AE) profile of volasertib is generally manageable and that volasertib may have antitumor activity. In a phase I study, reversible hematologic AEs (neutropenia, thrombocytopenia) constituted the dose-limiting toxicities (DLTs) in patients with progressive advanced or metastatic solid tumors who received single-agent volasertib. The maximum tolerated dose (MTD) was 400 mg; however, 300 mg was considered to be the recommended dose for further clinical investigation based on overall tolerability. Encouraging signs of antitumor activity were seen in three patients with confirmed partial response (PR) of lesions in urothelial cancer, ovarian cancer, or melanoma, and 40 % of patients experienced stable disease (SD) [13].

Although antineoplastic platinum agents like cisplatin and carboplatin have already exhibited promising results in clinical trials, many patients are refractory or relapse quickly after treatment. Preclinical data show that the combination of BI 2536 with cisplatin may confer synergism versus either therapy alone [14], which may be the result of a sensitization to cisplatin with Plk1 suppression [15]. The combination of BI 2536 and cisplatin resulted in improved efficacy in both in vitro and murine xenograft models compared with cisplatin alone [16]. A similar synergistic effect has also been observed following Plk1 downregulation in combination with carboplatin treatment in preclinical studies [17].

This phase I, dose-escalation study was conducted to determine the MTD of volasertib in combination with cisplatin or carboplatin and to evaluate the safety and activity of this combination in patients with advanced or metastatic solid tumors.

## Material and methods

### Trial design

This was a phase I, open-label, parallel-group, 3 + 3 dose-escalation trial of combination therapy with volasertib and cisplatin, or volasertib and carboplatin, conducted at two centers in Belgium (ClinicalTrials.gov ID: NCT00969761; 1230.6). The primary endpoint was determination of the MTD, defined as the highest dose of volasertib in combination with cisplatin or carboplatin at which the incidence of DLTs during the first cycle was less than 33 % (i.e., fewer than two of six patients). Secondary endpoints included pharmacokinetics and evaluation of overall safety and antitumor activity. Safety endpoints included the incidence and intensity of AEs, DLTs, serious and significant AEs, laboratory parameters, and vital signs. Efficacy endpoints included overall response rate, duration of objective response, rate and duration of disease control, and progression-free survival (PFS).

### Patient selection

Patients aged ≥18 years with confirmed diagnosis of advanced, non-resectable or metastatic solid tumors, who had failed conventional treatment, or for whom no therapy of proven efficacy existed, or who were not amenable to established forms of treatment, were eligible for this trial. Additional inclusion criteria were: indication for treatment with platinum therapy as judged by the investigator; Eastern Cooperative Oncology Group Performance Status (ECOG PS) ≤2; and recovery from Common Terminology Criteria for Adverse Events (CTCAE) grade 2 to 4 therapy-related AEs from previous systemic anticancer therapies or radiotherapies (except alopecia of CTCAE grade 2). Patients were excluded if they had clinical evidence of symptomatic progressive brain or leptomeningeal disease during the past 6 months; second malignancy currently requiring another anticancer therapy; absolute neutrophil count (ANC) <1,500/mm^3^; platelet count <100,000/mm^3^; serum creatinine >1.5 mg/dL (>132 μM/L, SI unit equivalent) or creatinine clearance <70 mL/min (as calculated according to Cockcroft–Gault formula for glomerular filtration rate [GFR] estimate); known history of relevant QT prolongation, (e.g., long QT syndrome); pre-existing clinically relevant hearing loss; treatment with other investigational drugs or participation in another clinical interventional trial within the 4 weeks prior to the start of therapy or concomitantly with this trial, or systemic anticancer therapy or radiotherapy within the 4 weeks prior to the start of therapy or concomitantly with this trial, with the exception of steroids and bisphosphonates. The study was conducted in accordance with the ethical principles originating from the Declaration of Helsinki and Good Clinical Practice as defined by the International Conference on Harmonization. The study was approved by the local Independent Ethics Committees and/or Institutional Review Boards of the participating centers and the Federal Agency for Medicines and Health Products, Brussels, Belgium. All participating patients gave written informed consent.

### Treatment

Volasertib was administered as a single dose by intravenous infusion over 2 hours, starting in the first treatment cycle, on day 1 every 3 weeks, given 30 minutes after a 1-hour intravenous cisplatin infusion on day 1 of a 3-week cycle or a 1-hour intravenous carboplatin infusion on day 1 of a 3-week cycle (Fig. S1 [Online Resource 1]). The starting doses for each platinum combination were volasertib 100 mg combined with cisplatin 60 mg/m^2^ or carboplatin area under the concentration versus time curve (AUC)4. Subsequent dose cohorts are listed in Table S1 (Online Resource 2). Target doses for carboplatin were calculated using the Calvert formula [18] to achieve AUC4, AUC5, and AUC6: dose (mg) = target AUC x (GFR + 25); GFR = (140 – age [years]) x (actual weight [kg])/(72 x serum creatinine [mg/dL]); multiplied by another factor of 0.85 if female. The maximum absolute dose of carboplatin per cycle was limited to 900 mg, as recommended by the US Food and Drug Administration [19].

Dose escalation of volasertib followed a 3 + 3 design, whereby cohorts of three to six patients were entered sequentially. The first patient of each dosage cohort was treated and observed until day 15 before the remaining two patients were entered. The decision regarding dose escalation was based on the occurrence of DLTs during the first treatment cycle. After determination of the MTD, patient enrollment at higher dosage tiers was suspended. Up to 12 patients could be treated at the MTD. This volasertib dose-escalation scheme was applied to each platinum combination. Volasertib plus cisplatin or carboplatin were given for up to six cycles; volasertib monotherapy was continued after six cycles of combination therapy until progression or intolerance. Dose reductions were permitted for patients with DLTs. Upon development of a DLT, study treatment was stopped temporarily and could be resumed (after recovery, with a maximum of 35 days between two dose administrations) at a reduced dose according to prespecified dose-reduction schemes for non-hematologic and hematologic AEs.

### Assessments

All patients were monitored carefully for AEs during and after treatment until discontinuation from trial. AEs were documented and graded according to CTCAE version 3.0 and assessed for relatedness to the combination treatment. DLTs were defined as any of the following AEs: drug-related grade 3 or 4 non-hematologic AEs (except ototoxicity and vomiting or diarrhea responding to supportive treatment); drug-related grade 4 neutropenia lasting ≥7 days and/or complicated by infection; grade 4 thrombocytopenia; or drug-related grade 3 febrile neutropenia (ANC <1,000/mm^3^ and fever ≥38.5 °C).

Blood was collected at specified time points during the first and second cycles of each treatment schedule for pharmacokinetic analyses to determine the plasma concentration of volasertib and/or total platinum. Plasma concentrations in cycle 1 were determined before the start of platinum infusions (–1 hour 35 minutes relative to the start of the volasertib infusion), shortly before the end of platinum infusion (–30 minutes), during volasertib infusion (1 hour), immediately before the end of volasertib infusion (2 hours), and at 3, 8, 24, 48, 168, and 336 hours after the start of volasertib infusion. The plasma concentrations in cycle 2 were determined before the start of platinum infusions (–1 hour 35 minutes), shortly before end of platinum infusion (–30 minutes), and immediately before the end of volasertib infusion (2 hours). Plasma concentrations of both volasertib and CD 10899, the predominant circulating hydroxylated metabolite of volasertib previously identified in early clinical studies of volasertib metabolism in cancer patients [13], were determined simultaneously by validated high performance liquid chromatography-tandem mass spectrometry (HPLC-MS/MS) assay using [D_3_]volasertib and [D_3_]CD 10899 as internal standards. The samples were subjected to solid-phase extraction in a 96-well plate format. Chromatography was achieved on an analytical reversed-phase HPLC column with gradient elution. The substances were detected and quantified by HPLC-MS/MS using electrospray ionization in the positive ion mode. Assay performance during the study was assessed by back-calculation of calibration standards, tabulation of the standard curve fit function parameters and measurement of quality control samples. No relevant interference of endogenous compounds was observed in human plasma samples. The calibration curves were linear over the range of concentrations from 0.200 to 200 ng/mL volasertib base salt (BS) and CD 10899 BS using a plasma volume of 50 μL. Plasma concentrations of cisplatin and carboplatin were determined as total platinum by inductively coupled plasma-mass spectrometry (ICP-MS) using ^175^Lu (lutetium) as an internal standard. Samples were diluted and acidified prior to analysis and were introduced into the ICP-MS system without further sample preparation. The ions were separated and detected in the mass spectrometer and the peak areas of platinum and lutetium were determined.

Tumor measurements were performed at screening and at the end of every other treatment cycle by computed tomography or magnetic resonance imaging. Overall response was assessed according to Response Evaluation Criteria In Solid Tumors (RECIST; version 1.1) [20].

### Statistical analysis

This was an open-label study and all analyses were descriptive and exploratory. The analysis population was the treated set that consisted of all patients who received ≥1 administration of volasertib with cisplatin or carboplatin. The analysis of the primary endpoint, determination of the MTD, was performed on the basis of DLT observed during the first cycle, per dose cohort. The treated set was used for tumor response and pharmacokinetic analyses.

## Results

### Patient demographics and disposition

In total, 61 patients received volasertib in combination with either cisplatin (*n* = 30) or carboplatin (*n* = 31). Patient demographics are shown in Table 1. In the volasertib/cisplatin arm, the median age (range) was 55 (17–77) years and 53.3 % were male. All patients had an ECOG PS of 0 (43.3 %) or 1 (56.7 %). Four of 30 patients (13.3 %) in the volasertib/cisplatin arm discontinued the trial before starting cycle 2 (progressive disease [PD], *n* = 2 [6.7 %]; DLT, *n* = 1 [3.3 %]; other AE [neoplasm progression], *n* = 1 [3.3 %]). Twenty-six patients (86.7 %) received at least two cycles of combination treatment. Of these 26 patients, two patients (7.7 %) discontinued the trial because of other AEs, while 24 patients (92.3 %) continued their treatment until PD.

**Table 1:** Patient demographics and baseline characteristics (treated set)

	Volasertib/cisplatin (*n* = 30)	Volasertib/carboplatin (*n* = 31)
Age, median (range), years	55 (17–77)	58 (23–81)
Male/female, *n* (%)	16 (53.3)/14 (46.7)	18 (58.1)/13 (41.9)
Baseline ECOG PS, *n* (%)		
0	13 (43.3)	14 (45.2)
1	17 (56.7)	17 (54.8)
Stage at diagnosis, *n* (%)		
0	0 (0.0)	1 (3.2)
I	1 (3.3)	3 (9.7)
II	1 (3.3)	3 (9.7)
III	9 (30.0)	8 (25.8)
IV	15 (50.0)	15 (48.4)
Unknown	4 (13.3)	1 (3.2)
Type of cancer, *n* (%)^a^		
NSCLC	8 (26.7)	6 (19.4)
CRC	4 (13.3)	4 (12.9)
Soft tissue sarcoma	4 (13.3)	4 (12.9)
Melanoma	3 (10.0)	0 (0.0)
Biliary tree	2 (6.7)	0 (0.0)
Bladder	0 (0.0)	3 (9.7)
Breast	0 (0.0)	2 (6.5)
Pleura	0 (0.0)	2 (6.5)
Any prior anticancer therapy, *n* (%)		
Systemic chemotherapy	28 (93.3)	29 (93.5)
Surgery	16 (53.3)	19 (61.3)
Radiotherapy	19 (63.3)	15 (48.4)
Other	14 (46.7)	15 (48.4)

Abbreviations: *CRC* colorectal cancer, *ECOG PS* Eastern Cooperative Oncology Group Performance Status, *NSCLC* non-small cell lung cancer

^a^In >5 % of patients

In the volasertib/carboplatin arm, the median age (range) was 58 (23–81) years and 58.1 % were male. All patients had an ECOG PS of 0 (45.2 %) or 1 (54.8 %). Three out of 31 patients (9.7 %) in the volasertib/carboplatin arm discontinued the trial before starting cycle 2 (PD, *n* = 2 [6.5 %]; refusal to continue receiving trial medication, *n* = 1 [3.2 %]). Twenty-eight patients (90.3 %) received at least two cycles of combination treatment. Of these 28 patients, three patients (10.7 %) discontinued the trial because of other AEs, while 25 patients (89.3 %) continued their treatment until PD.

### Treatment exposure

In the volasertib/cisplatin arm, the median (range) number of treatment cycles of volasertib administered overall was 3.5 (1–20), with a total absolute dose exposure to volasertib across the cohorts of 675.0 (200–4,400) mg. The median (range) number of treatment cycles of cisplatin in combination with volasertib administered overall was 3.5 (1–6) and the median (range) total absolute dose exposure to cisplatin across the cohorts was 440.5 (130–1,200) mg.

In the volasertib/carboplatin arm, the median (range) number of treatment cycles of volasertib administered overall was 2.0 (1–14), with a total absolute dose exposure to volasertib across the cohorts of 600.0 (200–3,300) mg. The median number (range) of treatment cycles of carboplatin in combination with volasertib administered overall was 2.0 (1–6) and the median (range) total absolute dose exposure to carboplatin across the cohorts was 1647.0 (426–4,382) mg.

### Primary endpoint: determination of MTD assessed by DLTs in cycle 1

In the volasertib/cisplatin arm, no DLTs were observed during the first treatment cycle in the first four cohorts tested (volasertib/cisplatin: 100/60, 100/75, 200/75 and 300/75; Table 2). One of six patients in the 300/100 cohort experienced a DLT during cycle 1 (grade 4 neutropenia for ≥7 days). Dose escalation to 350/75 resulted in two of six patients experiencing a DLT during cycle 1 (grade 3 increased alanine aminotransferase [ALT; *n* = 1]; grade 3 fatigue and grade 4 neutropenia for ≥7 days [*n* = 1]). As there were two patients with DLTs during the first cycle with the 350/75 dose combination of six patients, this appeared to be above the MTD. The combination of volasertib 300 mg and cisplatin 100 mg/m^2^ was determined to be the MTD. Two of the six patients in the extension cohort at the MTD experienced a DLT during cycle 1 (grade 3 blood creatinine increased [*n* = 1]; grade 3 fatigue [*n* = 1]).

**Table 2:** Overall summary of DLTs occurring in cycle 1 (treated set)

Dose cohorts		*N*	*n* with DLTs	DLT
Volasertib (mg)/Cisplatin (mg/m^2^)	100/60	3	0	None
	100/75	3	0	None
	200/75	3	0	None
	300/75	3	0	None
	300/100^a^	6	1	Grade 4 neutropenia for ≥7 days
	300/100^b^	6	2	Grade 3 increased blood creatinine (*n* = 1); grade 3 fatigue (*n* = 1)
	350/75	6	2	Grade 3 increased ALT (*n* = 1); grade 3 fatigue and grade 4 neutropenia for ≥7 days (*n* = 1)
Volasertib (mg)/Carboplatin (AUC)	100/4	3	0	None
	100/5	3	0	None
	200/5	3	0	None
	300/5	6	1	Grade 4 thrombocytopenia and grade 4 neutropenia for ≥7 days
	300/6^a^	6	1	Grade 4 thrombocytopenia
	300/6^b^	7^c^	1	Grade 4 thrombocytopenia
	350/5	3	2	Grade 4 thrombocytopenia (*n* = 1); grade 4 neutropenia for ≥7 days, grade 4 thrombocytopenia, grade 3 fatigue, grade 3 febrile neutropenia, grade 3 nausea, and grade 3 anorexia (*n* = 1)

Abbreviations: *ALT* alanine aminotransferase, *AUC* area under the concentration versus time curve, *DLTs* dose-limiting toxicities, *MTD* maximum tolerated dose

^a^Defined as the MTD

^b^MTD cohorts were expanded to further characterize safety

^c^One patient was not evaluable for MTD and was replaced

In the volasertib/carboplatin arm, no DLTs were observed during the first treatment cycle in the first three cohorts tested (100/AUC4, 100/AUC5, and 200/AUC5; Table 2). One of six patients enrolled in the 300/AUC5 cohort experienced two DLTs during the first treatment cycle (grade 4 thrombocytopenia and grade 4 neutropenia for ≥7 days). No additional DLTs were observed and the dose was escalated to 300/AUC6. One of the first six patients in the 300/AUC6 cohort experienced a DLT during cycle 1 (grade 4 thrombocytopenia). With dose escalation to 350/AUC5, two of three patients experienced DLTs in cycle 1 (grade 4 thrombocytopenia [*n* = 1]; grade 4 neutropenia for ≥7 days, grade 4 thrombocytopenia, grade 3 fatigue, grade 3 febrile neutropenia, grade 3 nausea, and grade 3 anorexia [*n* = 1]). The dose of 350/AUC5 was, therefore, considered above the MTD and the combination of volasertib 300 mg and carboplatin AUC6 was determined to be the MTD. One patient in the next six patients in the extension cohort at the MTD experienced a DLT (grade 4 thrombocytopenia) in cycle 1.

### Safety

DLTs after the first cycle were experienced by a total of three patients in the volasertib/cisplatin arm. Two patients treated with 300/75 experienced DLTs: one had grade 4 neutropenia for ≥7 days in cycle 3 and grade 3 febrile neutropenia in cycle 4, and a second experienced grade 4 neutropenia for ≥7 days in cycle 3. One patient treated with 300/100 experienced grade 4 neutropenia for ≥7 days in cycle 4. No patients in the volasertib/carboplatin arm experienced a DLT after the first cycle.

All 30 patients in the volasertib/cisplatin arm had ≥1 AE regardless of CTCAE grade and relatedness. All of the patients in this treatment arm also had ≥1 drug-related AE. The most common drug-related AEs across the dose cohorts and for all grades were anemia, neutropenia, leukopenia, nausea, vomiting, and thrombocytopenia (Table 3). Drug-related grade 3/4 AEs occurred in every dose cohort in the volasertib/cisplatin arm and in 21 patients (70.0 %) overall. The most common drug-related grade 3/4 AEs were neutropenia, lymphopenia, leukopenia, fatigue, and thrombocytopenia. Thirteen patients (43.3 %) had a total of 27 serious adverse events (SAEs) during the treatment period. Of these, 12 drug-related SAEs experienced by seven patients included nausea, vomiting, and increased blood creatinine (two patients, 6.7 % for each event). Other drug-related SAEs were single cases of anemia, neutropenia, febrile neutropenia, pyrexia, tumoral hemothorax, and orthostatic hypotension. There were no deaths in the volasertib/cisplatin arm during treatment.

**Table 3:** Overall summary of drug-related AEs (treated set)^a^

Volasertib (mg)/cisplatin (mg/m^2^)	100/60 (*n* = 3)		100/75 (*n* = 3)		200/75 (*n* = 3)		300/075 (*n* = 3)		300/100 (*n* = 12)		350/75 (*n* = 6)		Total (*N* = 30)	
Grade	All	3/4	All	3/4	All	3/4	All	3/4	All	3/4	All	3/4	All	3/4
Anemia	3	0	3	1	1	0	3	0	8	2	4	0	22 (73.3)	3 (10.0)
Neutropenia	2	0	3	2	0	0	3	2	9	6	5	3	22 (73.3)	13 (43.3)
Leukopenia	2	0	3	0	0	0	3	1	9	3	4	2	21 (70.0)	6 (20.0)
Nausea	2	0	2	0	1	0	2	0	11	3	3	0	21 (70.0)	3 (10.0)
Vomiting	2	0	2	0	3	0	2	1	11	2	1	0	21 (70.0)	3 (10.0)
Thrombocytopenia	1	0	1	0	1	0	3	0	9	4	5	1	20 (66.7)	5 (16.7)
Fatigue	1	0	2	0	1	0	1	0	8	4	4	2	17 (56.7)	6 (20.0)
Lymphopenia	1	1	2	1	2	1	1	0	7	4	3	2	16 (53.3)	9 (30.0)
Decreased appetite	1	0	1	0	0	0	2	0	7	0	4	0	15 (50.0)	0 (0.0)
Increased blood creatinine	1	0	1	0	0	0	0	0	4	1	0	0	6 (20.0)	1 (3.3)
Peripheral sensory neuropathy	0	0	2	0	0	0	1	0	2	0	1	0	6 (20.0)	0 (0.0)
Tinnitus	0	0	1	0	0	0	0	0	5	0	0	0	6 (20.0)	0 (0.0)
Alopecia	0	-	0	-	0	-	2	-	0	-	2	-	4 (13.3)	-
Stomatitis	0	0	0	0	0	0	1	0	2	0	1	0	4 (13.3)	0 (0.0)
Constipation	0	0	0	0	0	0	0	0	2	0	1	0	3 (10.0)	0 (0.0)
Diarrhea	0	0	1	0	0	0	0	0	2	0	0	0	3 (10.0)	0 (0.0)
Volasertib (mg)/carboplatin (AUC)	100/4 (*n* = 3)		100/5 (*n* = 3)		200/5 (*n* = 3)		300/5 (*n* = 6)		300/6 (*n* = 13)		350/5 (*n* = 3)		Total (*N* = 31)	
Grade	All	3/4	All	3/4	All	3/4	All	3/4	All	3/4	All	3/4	All	3/4
Anemia	2	0	2	0	2	0	6	2	13	3	3	3	28 (90.3)	8 (25.8)
Thrombocytopenia	1	0	2	1	2	1	5	3	12	8	3	2	25 (80.6)	15 (48.4)
Leukopenia	1	0	1	0	2	0	5	3	11	3	2	2	22 (71.0)	8 (25.8)
Neutropenia	0	0	1	1	1	0	5	4	12	8	3	3	22 (71.0)	16 (51.6)
Lymphopenia	1	0	1	0	2	0	6	2	8	3	1	0	19 (61.3)	5 (16.1)
Fatigue	1	0	1	1	0	0	2	0	7	0	2	1	13 (41.9)	2 (6.5)
Nausea	0	0	0	0	0	0	3	0	6	0	1	1	10 (32.3)	1 (3.2)
Vomiting	0	0	0	0	1	0	2	0	5	0	1	0	9 (29.0)	0 (0.0)
Decreased appetite	0	0	1	0	0	0	0	0	4	0	1	1	6 (19.4)	1 (3.2)
Increased blood creatinine	0	0	0	0	1	0	1	0	2	0	0	0	4 (12.9)	0 (0.0)

Abbreviations: *AEs* adverse events, *AUC* area under the concentration versus time curve, ^a^AEs (all grade) occurring in ≥10 % of patients

Two patients (6.7 %) in the volasertib/cisplatin arm discontinued trial drug because of AEs: one patient in the 200/75 cohort had cisplatin discontinued because of a grade 2 hypersensitivity reaction during the sixth cycle, and one patient in the 300/100 had both volasertib and cisplatin discontinued after the first cycle because of a DLT of grade 3 increased blood creatinine. Six patients (20.0 %) had a total of 10 AEs that led to dose reductions of one or both drugs. One patient (33.3 %) in the 100/75 cohort had a reduction of cisplatin only to 60 mg/m^2^ starting in cycle 4 following grade 3 neutropenia. One patient (33.3 %) in the 300/75 cohort had dose reductions of both drugs and was treated with 300/60 in cycle 5 and 200/60 in cycle 6 due to grade 4 neutropenia. This patient continued treatment with volasertib for a total of 20 cycles. Three patients (25.0 %) in the 300/100 cohort had a dose reduction to 200/75 in cycle 2 (grade 4 neutropenia and grade 3 thrombocytopenia [*n* = 1]; grade 3 fatigue [*n* = 2]). One patient (16.7 %) in the 350/75 cohort had a dose reduction to 300/60 in cycle 2 due to grade 4 neutropenia, grade 3 thrombocytopenia, grade 3 fatigue, and grade 1 pyrexia.

All 31 patients in the volasertib/carboplatin arm had ≥1 AE regardless of CTCAE grade and relatedness. Thirty patients (96.8 %) in the volasertib/carboplatin arm had ≥1 drug-related AE (Table 3). The most common drug-related AEs across the dose cohorts were anemia, thrombocytopenia, leukopenia, neutropenia, and lymphopenia. Drug-related grade ≥3 AEs occurred in 23 patients (74.2 %) overall. The most common drug-related grade ≥3 AEs were neutropenia, thrombocytopenia, anemia, and leukopenia. Eleven patients (35.5 %) had a total of 37 SAEs during the treatment period. Of these, 13 drug-related SAEs experienced by three patients included anemia, neutropenia, and thrombocytopenia (9.7 % with each event), and single cases of decreased appetite, nausea, fatigue, and febrile neutropenia. Two patients in the volasertib/carboplatin arm (both in the 300/AUC6 cohort) died while on treatment. One patient died of respiratory tract infection and septic shock after seven cycles of volasertib and six cycles of carboplatin (normal neutrophil count on the day of death), and one patient died of neoplasm progression after 11 cycles of volasertib and six cycles of carboplatin (during the follow-up period after discontinuation for progression). Neither of the fatal SAEs was considered to be drug related.

Four patients (12.9 %) in the volasertib/carboplatin arm had a total of six AEs that led to dose reductions of one or both drugs. One patient (16.7 %) in the 300/AUC5 cohort had a dose reduction of both drugs and was treated with 200/AUC4 starting in cycle 2 due to grade 4 neutropenia and grade 4 thrombocytopenia. This patient continued treatment with volasertib for a total of 14 cycles. Two patients (15.4 %) in the 300/AUC6 cohort had dose reductions (dose reduction of carboplatin only in one case). One patient was treated with 300/AUC4 starting in cycle 4 due to grade 3 thrombocytopenia; the second patient received 200/AUC4 starting in cycle 2 due to grade 4 thrombocytopenia (both dose reductions were protocol violations; per protocol, these two patients should have received 200/AUC5). One patient (33.3 %) in the 350/AUC5 cohort was reduced to 300/AUC4 in cycle 2 due to grade 4 neutropenia and grade 4 thrombocytopenia.

### Pharmacokinetics

**Fig. 1:**
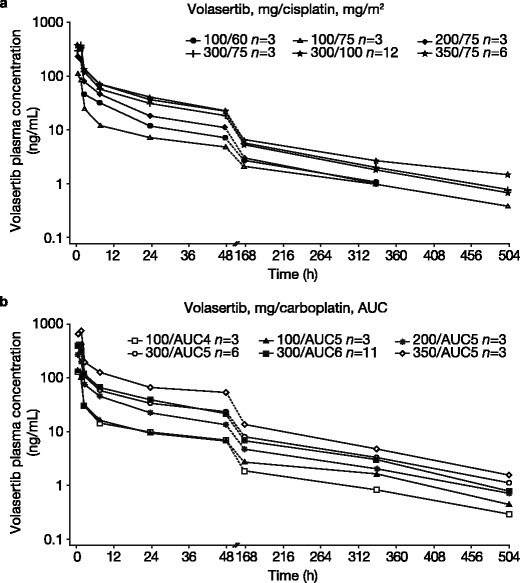
gMean plasma concentration-time profiles of total volasertib after intravenous infusion of volasertib in combination with (**a**) cisplatin or (**b**) carboplatin (semi-log scale). Abbreviations: *AUC* area under the concentration versus time curve, *gMean* geometric mean

A total of 60 patients in the treated set were included in the pharmacokinetic set. One patient from the volasertib/carboplatin (300/AUC6) cohort was not included because the administration date and/or time of volasertib dosing was missing. Volasertib exhibited multi-exponential pharmacokinetic behavior with fast distribution after the end of infusion, followed by several slower elimination phases in combination with cisplatin (Fig. 1a) or carboplatin (Fig. 1b). Key pharmacokinetic parameters from both treatment arms were generally comparable with a high volume of distribution, moderate total plasma clearance and long t_1/2_ (Table 4). Dose-normalized pharmacokinetic exposure parameters of volasertib were similar with either cisplatin or carboplatin co-administration (Table 4).

**Table 4:** Overall summary of non-compartmental pharmacokinetic parameters of volasertib combined with cisplatin or carboplatin

	Volasertib/cisplatin			Volasertib/carboplatin		
	*n*	gMean	gCV (%)	*n*	gMean	gCV (%)
Volasertib						
AUC_0-∞,norm_ ([ng · h/mL]/mg)	28	16.7	30.4	28	19.3	26.6
C_max,norm_([ng/mL]/mg)	30	1.29	61.5	29	1.40	46.7
t_1/2_ (h)	28	137	26.0	28	166	32.0
CL (mL/min)	28	999	30.4	28	865	26.6
V_ss_ (L)	28	6780	53.6	28	7550	44.5
CD 10899^a^						
RAUC_0-∞,M/P_ (%)	26	21.9	33.9	26	18.6	27.1
t_1/2_ (h)	26	134	28.4	27	152	33.2

Abbreviations: *AUC*_*0-∞*_ area under the concentration-time curve in plasma over the time interval from 0 extrapolated to infinity, *CL* total clearance, *C*_*max*_ maximum measured concentration in plasma, *gCV* geometric coefficient of variation, *gMean* geometric mean, *norm* dose normalized, *RAUC*_*0-∞,M/P*_ AUC ratio metabolite CD 10899/volasertib, *t*_*1/2*_ terminal half-life, *V*_*ss*_ apparent volume of distribution at steady state

^a^Metabolite of volasertib

The area under the concentration-time curve in plasma over the time interval from 0 extrapolated to infinity (AUC_0–∞_) of CD 10899, volasertib’s primary metabolite, was approximately 20 % that of volasertib, independent of whether volasertib was combined with cisplatin or carboplatin (Table 4). CD 10899 showed similar pharmacokinetic behavior to volasertib. The overall geometric mean (gMean) half-lives of CD 10899 following intravenous infusion of volasertib were similar in the cisplatin and carboplatin arms (Table 4).

Both cisplatin and carboplatin exhibited multi-exponential disposition pharmacokinetics with a fast distribution phase after the intravenous infusion (data not shown). Total platinum plasma clearance was about 9.7 mL/min for cisplatin and 80.5 mL/min for carboplatin. Total platinum distributed in a small volume of around 73.2 L with cisplatin and around 196.2 L with carboplatin. Mean apparent half-lives of total platinum were 88.9 hours for cisplatin and 40.3 hours for carboplatin.

### Antitumor activity

Tumor response according to RECIST was evaluable in 26 of the 30 patients in the volasertib/cisplatin arm (Table 5). Four patients (13.3 %) did not have any post-baseline tumor assessments and were not evaluable for response. Best overall response (BOR) was PRs in two patients (6.7 %). One responder (100/75 cohort) was a 50-year-old female patient with an undifferentiated follicular dendritic reticulum cell sarcoma of the palatine tonsil. At screening, she had metastases in the lung. Prior to enrollment, she had received combination cyclophosphamide, vincristine, doxorubicin, and prednisone (BOR was PR). In this study, she received six cycles of cisplatin and 15 cycles of volasertib and achieved a PR after 43 days from treatment initiation and a PFS of 340 days. The second responder (300/75 cohort) was a 42-year-old female patient with a well differentiated follicular dendritic reticulum cell retroperitoneal sarcoma. She had metastases in the bone, liver, and muscles at screening. She had previously undergone surgery followed by combination cyclophosphamide, vincristine, doxorubicin, and prednisolone (BOR was PD). She received six cycles of cisplatin and 20 cycles of volasertib and achieved a PR after 78 days and a PFS of 436 days.

**Table 5:** Antitumor activity in evaluable patients

	Volasertib/cisplatin (*n* = 30)	Volasertib/carboplatin (*n* = 31)
Disease control rate, *n* (%)	13 (43.3)	8 (25.8)
PR confirmed	2 (6.7)^a^	2 (6.5)^b^
SD	11 (36.7)^c^	6 (19.4)^d^
PD	13 (43.3)	18 (58.1)
Missing^e^	4 (13.3)	4 (12.9)
Not evaluable	0 (0)	1 (3.2)
Median PFS, days (range)	93.5 (1–436)	43.0 (1–331)

Abbreviations: *CRC* colorectal cancer, *NSCLC* non-small cell lung cancer, *PD* progressive disease, *PFS* progression-free survival, *PR* partial response, *SD* stable disease

^a^Tumor types: follicular dendritic reticulum cell carcinoma of the palatine tonsil (*n* = 1), follicular dendritic reticulum cell retroperitoneal sarcoma (*n* = 1)

^b^Tumor types: hypopharynx carcinoma (*n* = 1), NSCLC (*n* = 1)

^c^Tumor types: NSCLC (*n* = 3), CRC (*n* = 3), melanoma (*n* = 2), bladder cancer (*n* = 1), breast cancer (*n* = 1), endocrine cancer (*n* = 1)

^d^Tumor types: NSCLC (*n* = 2), biliary tree cancer (*n* = 1), liver cancer (*n* = 1), pancreatic cancer (*n* = 1), pleural cancer (*n* = 1)

^e^Missing indicates that there was no tumor assessment post-baseline and response status could not be assessed

An additional 11 patients (36.7 %) in the volasertib/cisplatin arm achieved SD including the following tumor types: non-small cell lung cancer (NSCLC; *n* = 3), colorectal cancer (CRC; *n* = 3), and melanoma (*n* = 2). Disease control (defined as a BOR of SD, PR, or complete response) was achieved in 13 patients (43.3 %), with a median duration (defined as the time from the start of study treatment to the time of disease progression or death) of 155.0 (range, 97–436) days. Median PFS for all patients across the cohorts was 93.5 (range, 1–436) days.

In the volasertib/carboplatin arm, tumor response was evaluable in 26 of the 31 patients (Table 5). Four patients (12.9 %) did not have any post-baseline tumor assessments and were not evaluable for response, and one patient (3.2 %) had a non-evaluable tumor at baseline. BOR was PRs in two patients (6.5 %). One responder (300/AUC5 cohort) was a 65-year-old male patient with a poorly differentiated hypopharynx carcinoma. He had metastases in the lung at screening. Prior to enrollment, he had previously received combination chemotherapy with cisplatin and fluorouracil, followed by combination carboplatin and fluorouracil (BOR was PR) and sequential single-agent therapy with methotrexate (BOR was SD), docetaxel (BOR was SD), and zalutumumab. He received six cycles of carboplatin and 14 cycles of volasertib and achieved a PR 50 days after treatment initiation and a PFS of 331 days. The second responder (300/AUC6 cohort) was a 60-year-old male patient with squamous NSCLC. He had metastases in the lung and was stage IV at screening. He had previously received a platinum doublet with cisplatin and gemcitabine (BOR was SD), followed by sequential single-agent therapy with pemetrexed (BOR was PD), docetaxel (BOR was PD), and an investigational survivin inhibitor (BOR was PD). He received six cycles of carboplatin and 11 cycles of volasertib, and achieved a PR after 37 days and a PFS of 243 days.

An additional six patients (19.4 %) in the volasertib/carboplatin arm achieved SD including two patients with NSCLC. Disease control was achieved in eight patients (25.8 %), with a median (range) duration of disease control overall of 183.5 (100–331) days. Median (range) PFS for all patients across the cohorts was 43.0 (1–331) days.

## Discussion

This was a phase I, open-label, dose-escalation study designed to determine the MTD, DLTs, safety, pharmacokinetics, and antitumor effects of the Plk inhibitor volasertib administered in combination with cisplatin or carboplatin in patients with advanced/metastatic solid tumors. The MTDs were determined to be volasertib 300 mg plus cisplatin 100 mg/m^2^ and volasertib 300 mg plus carboplatin AUC6 (limited to a maximum dose of 900 mg) administered on day 1 of a 3-week cycle. This MTD for volasertib combination therapy is the same as the recommended dose for volasertib monotherapy in solid tumors [13].

The most frequently observed DLTs were hematologic changes, which were expected based on previous experience with single-agent volasertib [13] and single-agent platinums [21, 22]. The AEs observed in this study were primarily a result of hematopoietic suppression from both agents in each combination. No additive effects were detected, indicating that each drug (volasertib, cisplatin, or carboplatin) can be administered in combination at the recommended maximum single-agent doses without an increase in AEs or unexpected safety findings. The higher frequency of hematologic AEs at volasertib doses exceeding 300 mg may be related to volasertib more than to the combination partner since comparable AE frequencies were observed with volasertib 300 mg combined with different doses of cisplatin or carboplatin. However, because separate causal assessments for volasertib and platinum were not performed, all observed AEs should be related to the combination and were not necessarily attributable to volasertib alone.

The current study of volasertib in combination with cisplatin or carboplatin showed no influence of cisplatin or carboplatin on the pharmacokinetics or metabolism of volasertib. As shown with monotherapy [13], volasertib exhibited multi-exponential pharmacokinetic behavior with extensive distribution into deep body compartments, a long t_1/2_ and moderate clearance. Based on a comparison with historical data for volasertib monotherapy, platinum drugs showed no influence on the pharmacokinetics of volasertib. Both cisplatin and carboplatin (co-administered with volasertib), measured as total platinum, showed multi-exponential pharmacokinetic behavior and the major pharmacokinetic parameters (maximum measured concentration in plasma [C_max_], AUC, and clearance) were comparable to the published data on monotherapy of the respective compounds [23–25].

In this population of patients with advanced/metastatic solid tumors, volasertib in combination with cisplatin or carboplatin showed encouraging signs of antitumor activity. Of 26 evaluable patients treated with volasertib/cisplatin, two had a PR and 11 achieved SD as best response. In combination with carboplatin, two of 26 evaluable patients had a PR and six achieved SD. One interesting finding was the activity observed in two female patients with follicular dendritic reticulum cell sarcoma, an orphan disease with sensitivity to drugs used for treatment of mesenchymal tumors and lymphoma. Both patients achieved durable PRs with volasertib/cisplatin treatment, with decreases in tumor size that persisted after completion of the maximum number of cycles with platinum.

It is generally acknowledged that novel agents/regimens for the treatment of patients with advanced/metastatic solid tumors are an unmet need. This trial demonstrated a generally acceptable safety profile and antitumor activity for volasertib in combination with cisplatin or carboplatin in patients with advanced/metastatic solid tumors. These data suggest that the investigation of volasertib for the treatment of patients with advanced/metastatic solid tumors, in particular, in combination with cytotoxic agents like platinums, is warranted. Additional studies focusing on predictive biomarkers would be beneficial to better understand the role of Plk inhibition in tumor development and anticancer therapy.

## Electronic supplementary material

Below is the link to the electronic supplementary material.

Supplementary Fig. 1 Infusion schemes for volasertib combined with (**a**) cisplatin or (**b**) carboplatin on day 1 of a 3-week cycle (PDF 476 kb)

ESM 2 (DOC 34 kb)
