# Supplementary material for: Phase I trial of volasertib, a Polo-like kinase inhibitor, plus platinum agents in solid tumors: safety, pharmacokinetics and activity
Source: Invest New Drugs. 2015 Mar 22;33(3):611–20. doi: 10.1007/s10637-015-0223-9 (PMC4435638; doi:10.1007/s10637-015-0223-9)
Supplement: Supplementary file 1 — Infusion schemes for volasertib combined with (a) cisplatin or (b) carboplatin on day 1 of a 3-week cycle (PDF 476 kb) [file 10637_2015_223_MOESM1_ESM.pdf]

Online Resource

Information for submission of supplementary material

Title: Phase I trial of volasertib, a Polo-like kinase inhibitor, plus platinum agents in solid tumors

Journal: Investigational New Drugs

Authors: Ahmad Awada · Herlinde Dumez · Philippe G. Aftimos · Jo Costermans · Sylvie Bartholomeus · Kathleen Forceville · Thierry Berghmans · Marie-Anne Meeus · Jessica Cescutti · Gerd Munzert · Korinna Pilz · Dan Liu · Patrick Schöffski

Corresponding author: Dr A. Awada; Institut Jules Bordet, Université Libre de Bruxelles, Boulevard de Waterloo 121, B-1000 Brussels, Belgium; E-mail: ahmad.awada@bordet.be; Phone: +32 2 541 31 89; Fax: +32 2 541 33 39

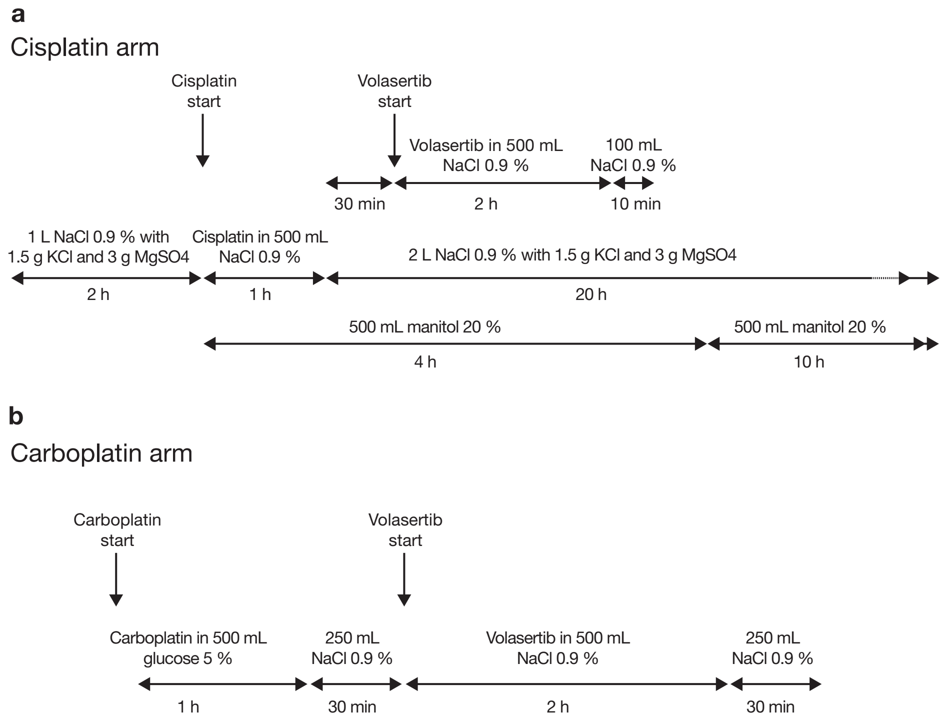
a
Cisplatin arm
Cisplatin
start
Volasertib
start
Volasertib in 500 mL
NaCl 0.9 %
100 mL
NaCl 0.9 %
30 min
2 h
10 min
1 L NaCl 0.9 % with
1.5 g KCl and 3 g MgSO4
Cisplatin in 500 mL
NaCl 0.9 %
2 L NaCl 0.9 % with 1.5 g KCl and 3 g MgSO4
2 h
1 h
20 h
500 mL manitol 20 %
500 mL manitol 20 %
4 h
10 h
b
Carboplatin arm
Carboplatin
start
Volasertib
start
Carboplatin in 500 mL
glucose 5 %
250 mL
NaCl 0.9 %
Volasertib in 500 mL
NaCl 0.9 %
250 mL
NaCl 0.9 %
1 h
30 min
2 h
30 min
