# Supplementary material for: Phase I trial of volasertib, a Polo-like kinase inhibitor, plus platinum agents in solid tumors: safety, pharmacokinetics and activity
Source: Invest New Drugs. 2015 Mar 22;33(3):611–20. doi: 10.1007/s10637-015-0223-9 (PMC4435638; doi:10.1007/s10637-015-0223-9)
Supplement: Supplementary file 2 — (DOC 34 kb) [file 10637_2015_223_MOESM2_ESM.doc]

**Online Resource 2**

**Supplementary Table 1.** Dosing cohorts to determine MTD for volasertib in combination with **(a)** cisplatin or **(b)** carboplatin. Abbreviations: *AUC* area under the concentration versus time curve, *MTD* maximum tolerated dose

**a**

| Cohort | Cisplatin (mg/m^2^ ) day 1 each cycle | Volasertib (mg)  day 1 each cycle |
| --- | --- | --- |
| Cohort A1 | 60 | 100 |
| Cohort A2 | 75 | 100 |
| Cohort A3 | 75 | 200 |
| Cohort A4 | 75 | 300 |
| Cohort A5 | 100 | 300 |
| Cohort A6 | 75 | 350 |

**b**

| Cohort | Carboplatin  day 1 each cycle | Volasertib (mg)  day 1 each cycle |
| --- | --- | --- |
| Cohort B1 | AUC4 | 100 |
| Cohort B2 | AUC5 | 100 |
| Cohort B3 | AUC5 | 200 |
| Cohort B4 | AUC5 | 300 |
| Cohort B5 | AUC6 | 300 |
| Cohort B6 | AUC5 | 350 |
